# The Prevalence and Clinical Implications of Rectal SARS-CoV-2 Shedding in Danish COVID-19 Patients and the General Population

**DOI:** 10.3389/fmed.2021.804804

**Published:** 2022-01-13

**Authors:** Julie Niemann Holm-Jacobsen, Caspar Bundgaard-Nielsen, Louise Søndergaard Rold, Ann-Maria Jensen, Shakil Shakar, Marc Ludwig, Karina Frahm Kirk, Mette Line Donneborg, Julia Helena Vonasek, Benjamin Pedersen, Louise Thomsen Schmidt Arenholt, Søren Hagstrøm, Peter Leutscher, Suzette Sørensen

**Affiliations:** ^1^Centre for Clinical Research, North Denmark Regional Hospital, Hjoerring, Denmark; ^2^Department of Clinical Medicine, Aalborg University, Aalborg, Denmark; ^3^Department of Emergency Medicine, Pandemic Unit, North Denmark Regional Hospital, Hjoerring, Denmark; ^4^Department of Internal Medicine, North Denmark Regional Hospital, Hjoerring, Denmark; ^5^Department of Emergency Medicine, North Denmark Regional Hospital, Hjoerring, Denmark; ^6^Department of Infectious Diseases, Aalborg University Hospital, Aalborg, Denmark; ^7^Department of Pediatrics, North Denmark Regional Hospital, Hjoerring, Denmark; ^8^Intensive Care Unit, North Denmark Regional Hospital, Hjoerring, Denmark; ^9^Department of Gynecology and Obstetrics, North Denmark Regional Hospital, Hjoerring, Denmark; ^10^Department of Pediatrics, Aalborg University Hospital, Aalborg, Denmark

**Keywords:** COVID-19, SARS-CoV-2, rectal shedding, viral shedding, feces

## Abstract

**Background:** SARS-CoV-2 has resulted in a global pandemic since its outbreak in Wuhan, 2019. Virus transmission primarily occurs through close contact, respiratory droplets, and aerosol particles. However, since SARS-CoV-2 has been detected in fecal and rectal samples from infected individuals, the fecal-oral route has been suggested as another potential route of transmission. This study aimed to investigate the prevalence and clinical implications of rectal SARS-CoV-2 shedding in Danish COVID-19 patients.

**Methods:** Hospitalized and non-hospitalized adults and children who were recently tested with a pharyngeal COVID-19 test, were included in the study. A rectal swab was collected from all participants. Hospitalized adults and COVID-19 positive children were followed with both pharyngeal and rectal swabs until two consecutive negative results were obtained. RT-qPCR targeting the envelope gene was used to detect SARS-CoV-2 in the samples. Demographic, medical, and biochemical information was obtained through questionnaires and medical records.

**Results:** Twenty-eight of 52 (53.8%) COVID-19 positive adults and children were positive for SARS-CoV-2 in rectal swabs. Seven of the rectal positive participants were followed for more than 6 days. Two of these (28.6%) continued to test positive in their rectal swabs for up to 29 days after the pharyngeal swabs had turned negative. Hospitalized rectal positive and rectal negative adults were comparable regarding demographic, medical, and biochemical information. Furthermore, no difference was observed in the severity of the disease among the two groups.

**Conclusions:** We provided evidence of rectal SARS-CoV-2 shedding in Danish COVID-19 patients. The clinical importance of rectal SARS-CoV-2 shedding appears to be minimal.

## Introduction

In December 2019, an outbreak with the novel coronavirus severe acute respiratory syndrome coronavirus 2 (SARS-CoV-2), occurred (1, 2). Since then, the virus has resulted in a global pandemic and has infected more than 240 million individuals and led to more than four and a half million deaths (3, 4). SARS-CoV-2 causes coronavirus disease 2019 (COVID-19) (1) characterized by diverse clinical manifestations ranging from asymptomatic to critical with multiple organ failure (5–7). Common symptoms include fever, cough, and fatigue, but symptoms such as dyspnea, headache, and gastrointestinal symptoms are also reported (8–10). Children often experience a milder course of COVID-19 compared to adults (5), where they often present asymptomatic or with symptoms such as fever and/or cough (11, 12).

SARS-CoV-2 is primarily transmitted from person to person through close contact, respiratory droplets, and aerosol particles (13–22). However, another mode of transmission being suggested is the fecal-oral transmission (23–27). The fecal-oral transmission is of particular interest as the angiotensin-converting enzyme 2 receptor that SARS-CoV-2 utilizes to enter the host cells (28) is highly expressed in the gastrointestinal system (29–31). In addition, several studies have confirmed the presence of SARS-CoV-2 in feces and rectal swabs from individuals infected with SARS-CoV-2. Studies have furthermore shown that some individuals continue to shed virus in the intestines after shedding in the respiratory tract has stopped (25, 27, 32). The infectious potential of fecal SARS-CoV-2 is, however, still unknown, and only a few studies have been able to isolate active SARS-CoV-2 from fecal samples (24, 26). Most of the studies investigating SARS-CoV-2 in feces or rectal swabs have been conducted in China, and to the authors' knowledge, no study has investigated it in a North European population.

Therefore, we aimed to investigate the proportion of COVID-19 patients in Denmark who shed SARS-CoV-2 from the intestines. Furthermore, we aimed to investigate the possible correlation between rectal shedding of SARS-CoV-2 and the severity of the disease.

## Materials and Methods

### Study Participants

From the 12th of June 2020 to the 28th of February 2021, hospitalized and non-hospitalized participants were included in the study. Adult hospitalized patients with suspicion of or confirmed COVID-19 infection (by pharyngeal testing) were recruited from the pandemic units at North Denmark Regional Hospital and Aalborg University Hospital. Hospitalized and non-hospitalized children with suspicion of or confirmed COVID-19 infection (by pharyngeal testing) were recruited from the departments of pediatrics at North Denmark Regional Hospital and Aalborg University Hospital. Non-hospitalized children were further recruited through advertisements on social media. Lastly, non-hospitalized adults who had been tested with a pharyngeal swab as a part of the national COVID-19 surveillance program were recruited from the COVID-19 test centers at North Denmark Regional Hospital and through advertisements on social media. Non-hospitalized adults were tested for a variety of reasons, including COVID-19 symptoms, close contact with infected individuals, prior to an appointment at the doctor or hospital, traveling, work, etc. (Supplementary Table 3). The inclusion criterium in the study was a recent pharyngeal swab as a part of the national COVID-19 surveillance program.

### Study Design

From the hospitalized adults and children, daily pharyngeal and rectal or fecal swabs were collected (henceforth referred to as rectal swabs). If either the pharyngeal or rectal swabs at discharge were positive for SARS-CoV-2 by reverse transcription-quantitative polymerase chain reaction (RT-qPCR), the participants were asked to continue the pharyngeal and rectal swab collection at home. Sample collection proceeded until two consecutive negative pharyngeal and rectal swabs were obtained.

Non-hospitalized participants only delivered a single rectal swab in addition to their pharyngeal swab. However, from the non-hospitalized children who tested positive for SARS-CoV-2 in either the pharyngeal or rectal swab, both sample types continued to be collected until two consecutive negative tests were obtained. This was to get a better representation of rectal SARS-CoV-2 shedding in children, since they often experience a mild disease course, and rarely are admitted to the hospital.

### Data Collection

Demographic information, including age, gender, height, weight, smoking status, alcohol consumption, occupation, and symptoms, was collected from questionnaires, while clinical and biochemical information was collected from medical records. In addition, questionnaires concerning present symptoms were collected at each sample collection. Study data were collected and managed using REDCap electronic data capture tools hosted at the North Denmark Region (33, 34).

### Sample Collection and SARS-CoV-2 Testing

Pharyngeal and rectal samples were collected using FLOQSwabs and stored in 1x phosphate-buffered saline at 5°C (short term) or −20°C (long term). Rectal swabs collected at the homes of participants were delivered within 72 h to the laboratory and were subsequently stored at 5°C (short term) or −20°C (long term). RNA was extracted with the use of the QIAamp Viral RNA Mini Kit (Qiagen, Cat. No. 52906) automated on a QIAcube (QIAGEN) according to the manufacturer's protocol. The presence of SARS-CoV-2 was detected by RT-qPCR with primers and probes targeting the envelope gene of SARS-CoV-2 (LightMix Modular SARS-CoV (COVID-19) E-gene, Roche, Cat. No 53-0776-96) using the qRT-PCR Brilliant III Probe Master Mix (Agilent, Cat. No. 600884). The thermocycling settings were as follows; initial reverse transcription for 5 min at 55°C, followed by 5 min at 95°C, 45 cycles of 5 s at 95°C, 22 s at 60°C, and 15 s at 72°C, and a final elongation step for 30 s at 40°C. Each sample was analyzed in duplicates. Two positive controls (a pool of RNA from previous patients tested positive for SARS-CoV-2 and an RNA positive control enclosed with the LightMix Modular SARS-CoV (COVID-19) E-gene, Roche kit), were included on each plate together with three no template controls. A sample was assessed as positive when at least one of the duplicates had a Ct-value <40.

### Statistical Analysis

Data analyses were performed using R version 4.0.5 (35) with RStudio IDE (36). For numeric data, normal distribution and variances were assessed using Shapiro-Wilk's test and Bartlett's test, respectively. Normal distributed data were compared using Student's *t*-test, whereas non-parametric data were compared using the Mann-Whitney-Wilcoxon test. Categorical data were compared using the two proportion z test or the chi-square test. A *p*-value < 0.05 was regarded as significant for all the statistical tests.

### Ethics Approval

The study was approved by the North Denmark Region Committee on Health Research Ethics (N-20200036) and reported to the Danish Data Protection Agency. Informed written consent was obtained from all participants and the legal guardians of the children.

## Results

### Prevalence of SARS-CoV-2 Rectal Shedding

In total, 219 non-hospitalized and 55 hospitalized participants were included in the study. Among the 219 non-hospitalized participants, 10 were positive for SARS-CoV-2 in the pharyngeal swabs (4.6%), and of these five were positive in the rectal swabs (50.0%) (Table 1). The non-hospitalized participants encompassed 211 adults and eight children. Of the 211 adults, nine were positive for SARS-CoV-2 in the pharyngeal swabs (4.3%), and of these four were positive for SARS-CoV-2 in the rectal swabs (44.4%). Of the eight children, one child was positive in both the pharyngeal and rectal swabs. Among the 55 hospitalized participants, 42 were positive for SARS-CoV-2 in the pharyngeal swabs (76.4%), and of these 23 were positive in the rectal swabs (54.8%) (Table 1). The hospitalized participants encompassed 52 adults and three children. Of the 52 adults, 41 were positive for SARS-CoV-2 in the pharyngeal swabs (78.8%). Thus 11 of the hospitalized adults turned out not to be infected with SARS-CoV-2. Of the 41 pharyngeal positive adults, 22 were positive for SARS-CoV-2 in the rectal swabs as well (53.7%). Of the three children, one child was positive both in the pharyngeal and rectal swabs. Rectal SARS-CoV-2 was not detected in any of the pharyngeal negative participants (Supplementary Tables 1–3). The pharyngeal positive and negative hospitalized adults were comparable regarding demographic and clinical characteristics (Supplementary Table 1). Demographic and clinical data for children and non-hospitalized adults are shown in Supplementary Tables 2, 3.

**Table 1:** Outline of the participants in the study.

	**Participants**	**Participants with positive pharyngeal swab**	**Participants with positive rectal swab**
	**N**	**N (% of participants)**	**N (% of participants/% of participants with a positive pharyngeal swab)**
Non-hospitalized participants	219	10 (4.6)	5 (2.3/50.0)
Hospitalized participants	55	42 (76.4)	23 (41.8/54.8)
Total	274	52 (19.0)	28 (10.2/53.8)

### Hospitalized Adult COVID-19 Patients With and Without Rectal Shedding of SARS-CoV-2

The hospitalized rectal positive and rectal negative adult COVID-19 patients were comparable regarding demographics, clinical characteristics, information from admission, vital signs, laboratory findings, and radiologic findings (Tables 2–4). No difference was seen in the severity of the disease between the two groups based on the WHO clinical progression score (37) and admission to the intensive care unit (Table 3; Supplementary Table 4).

**Table 2:** Demographic and clinical characteristics of hospitalized COVID-19 adult patients with positive and negative rectal swabs, respectively.

**Demographics**	**Positive rectal swab**	**Negative rectal swab**	***P*-value**
	***N* = 22 (53.7%)**	***N* = 19 (46.3%)**	
Age, years, median (CI)	72.5 (65.4–75.5)	68 (62.1–71.5)	0.10
Gender, N (%)			
Male	15 (68.2)	12 (63.2)	0.99
Female	7 (31.8)	7 (36.8)	0.99
BMI, mean (CI)	28.1 (25.9–30.3)	29.6 (27.3–31.9)	0.36
Living in a nursing home, N (%)	2 (9.1)	0 (0.0)	0.53
Smoking, N (%)			
Yes	0 (0.0)	3 (15.8)	0.18
No	8 (36.4)	8 (42.1)	0.96
Former	14 (63.6)	8 (42.1)	0.29
Alcohol consumption, N (%)			
More units/week than recommended^a^	2 (9.1)	2 (10.5)	1.00
Occupation, N (%)			
Healthcare	2 (9.1)	2 (10.5)	1.00
Educational sector	0 (0.0)	0 (0.0)	NA
Eldercare	0 (0.0)	0 (0.0)	NA
Children and adolescents	0 (0.0)	0 (0.0)	NA
Retired	16 (72.7)	12 (63.2)	0.75
Other	4 (18.2)	5 (26.3)	0.80
**Clinical characteristics**	**Positive rectal swab**	**Negative rectal swab**	***P*** **-value**
	***N*** **= 22 (53.7%)**	***N*** **= 19 (46.3%)**	
Intestinal disease, N (%)	5 (22.7)	1 (5.3)	0.26
Risk factors, N (%)			
Cardiovascular disease	15 (68.2)	12 (63.2)	0.99
Hypertension	13 (59.1)	11 (57.9)	1.00
Pulmonary disease	9 (40.9)	6 (31.6)	0.77
Asthma	4 (18.2)	1 (5.3)	0.43
COPD	5 (22.7)	3 (15.8)	0.87
Severe overweight (BMI > 30)	8 (36.4)	10 (52.6)	0.46
Cancer	5 (22.7)	4 (21.1)	1.00
Type 1 or 2 diabetes	3 (13.6)	4 (21.1)	0.83
Symptoms, N (%)			
Cough	18 (81.8)	17 (89.5)	0.80
Dyspnea	16 (72.7)	14 (73.7)	1.00
Fever	12 (54.5)	12 (63.2)	0.81
Gastrointestinal symptoms	13 (59.1)	8 (42.1)	0.44
Nausea	7 (31.8)	5 (26.3)	0.97
Vomiting	4 (18.2)	1 (5.3)	0.43
Stomach ache	6 (27.3)	5 (26.3)	1.00
Diarrhea	11 (50.0)	6 (31.6)	0.38
Sore throat	11 (50.0)	5 (26.3)	0.22
Affected taste or smell	10 (45.5)	6 (31.6)	0.56
Headache	7 (31.8)	6 (31.6)	1.00
No symptoms	0 (0.0)	0 (0.0)	NA
Vaccination, N (%)^b^			
Vaccinated with first dose	1 (6.7)	0 (0.0)	1.00
Fully vaccinated	0 (0.0)	0 (0.0)	NA

a *Alcohol consumption was assessed according to the recommendations made by the Danish Health Authority about the low-risk limit for women (7 units per week) and men (14 units per week). Intestinal disease includes Crohn's disease, diverticulitis, steatosis, bowel cancer, gastric bypass, and intestinal resection. Cardiovascular disease includes hypertension, transient cerebral ischemia, ischemic heart disease, non-STEMI coronary thrombosis, atrial fibrillation, hypercholesterolemia, arterial sclerosis, femoral bypass surgery, cardiac insufficiency, cerebral apoplexy, normal pressure hydrocephalus, and 3rd degree AV block with subsequent pacemaker implantation. Pulmonary disease includes asthma, chronic obstructive pulmonary disease, sleep apnea, and partial lung resection. Vaccination status was self-reported*.

b *Patients were excluded from the statistical analysis because of undetectable or missing values. For vaccination status statistical analyses were based on 15 rectal positive patients and 8 rectal negative patients. CI, Confidence interval; BMI, Body Mass Index; COPD, Chronic Obstructive Pulmonary Disease; NA, Not available*.

**Table 3:** Information from admission of COVID-19 adult patients with positive and negative rectal swabs, respectively.

**Information from the admission**	**Positive rectal swab**	**Negative rectal swab**	***P*-value**
	***N* = 22 (53.7%)**	***N* = 19 (46.3%)**	
Days from first positive pharyngeal test to sample collection, median (CI)	5.5 (4.76–9.88)	10 (8.47–12.6)	0.06
Days from admission to discharge, median (CI)	6 (6.24–14.5)^a^	6 (5.34–9.51)	0.81
Medical treatment, N (%)			
Antibiotics	14 (63.6)	8 (42.1)	0.29
Corticosteroids	16 (72.7)	14 (73.7)	1
Antiviral drugs	13 (59.1)	14 (73.7)	0.51
Drug trial^b^	7 (31.8)	4 (22.2)	0.75
Oxygen support (at inclusion/at the patients' worst), N (%)			
No oxygen support	11 (50.0)/7 (31.8)	12 (63.2)/3 (15.8)	0.60/0.41
Oxygen by mask or nasal prongs	11 (50.0)/9 (40.9)	7 (36.8)/12 (63.2)	0.60/0.27
Oxygen by NIV or high flow	0 (0.0)/3 (13.6)	0 (0.0)/3 (15.8)	NA/1.00
Intubation and mechanical ventilation	0 (0.0)/0 (0.0)	0 (0.0)/0 (0.0)	NA/NA
Mechanical ventilation or vasopressors	0 (0.0)/3 (13.6)	0 (0.0)/1 (5.3)	NA/0.71
Mechanical ventilation and vasopressors, dialysis or ECMO	0 (0.0)/0 (0.0)	0 (0.0)/0 (0.0)	NA/NA
Disease severity (at inclusion/at the patient's worst), N (%)			
Moderate	21 (95.5)/16 (72.7)	19 (100.0)/15 (78.9)	1.00^c^/0.86
Severe	1 (4.5)/4 (18.2)	0 (0.0)/3 (15.8)	
Dead	0 (0.0)/2 (9.1)	0 (0.0)/1 (5.3)	
Admitted to the ICU	5 (22.7)	2 (10.5)	0.54
Outcome within 60 days, N (%)			
Recovered	19 (86.4)	18 (94.7)	0.71
Not recovered	1 (4.5)	0 (0.0)	1
Died	2 (9.1)	1 (5.3)	1

*Antibiotics include trimethoprim, moxifloxacin, piperacillin/tazobactam, meropenem, sulfamethoxazole/trimethoprim, ciprofloxacin, clarithromycin, amoxicillin/clavulanic acid, gentamicin, penicillin, pivmecillinam, and ampicillin. Corticosteroids include prednisolone and dexamethasone. Antiviral drugs include remdesivir and aciclovir*.

a *One patient was still admitted at the time of data analysis and was not included in the statistical analysis of days from admission to discharge*.

b *Patients were excluded from the statistical analysis because of undetectable or missing values. For drug trial the statistical analysis was based on 22 rectal positive patients and 18 rectal negative patients. Disease severity is based on the WHO clinical progression score obtained at inclusion and at the patient's worst (Supplementary Table 4) (37)*.

c *Statistical analysis was based on the moderate and severe disease stages. CI, Confidence interval; NIV, Non-invasive ventilation; ECMO, Extra Corporeal Membrane Oxygenation; NA, Not available; ICU, Intensive care unit*.

**Table 4:** Vital signs, laboratory findings, and radiologic findings of hospitalized COVID-19 adult patients with positive and negative rectal swabs, respectively.

	**Positive rectal swab**	**Negative rectal swab**	***P*-value**
	***N* = 22 (53.7%)**	***N* = 19 (46.3%)**	
**Vital signs**			
PaO_2_, kPa, median (range)^a^	8.75 (5.4–12)	8.8 (4.7–15.1)	0.51
Peripheral oxygen saturation, %, median (range)	94 (90–100)	95 (89–99)	0.73
Temperature, °C, median (CI)	37.3 (37.2–38.1)	37 (37.0–38.0)	0.79
Systolic blood pressure, mm Hg, median (range)	136 (114–192)	134 (96–162)	0.24
**Laboratory findings**			
Leucocytes,10^9^/l, median (range)	6.1 (0.2–23.6)	8.6 (2.8–12.6)	0.83
Increased (>10.0), N (%)	6 (27.3)	6 (31.6)	1.00
Decreased (<3.5), N (%)	5 (22.7)	2 (10.5)	0.54
Platelets,10^9^/l, median (range)	196 (33–519)	249 (96–435)	0.48
Increased (>400), N (%)	2 (9.1)	2 (10.5)	1.00
Decreased (<145), N (%)	8 (36.4)	5 (26.3)	0.72
CRP, mg/l, median (range)^a^	76 (5.4–260)	69.5 (18–181)	0.91
Normal (<10 mg/l), N (%)	2 (9.5)	0 (0.0)	0.52
Mildly elevated (10–19 mg/l), N (%)	2 (9.5)	1 (5.6)	
Moderately elevated (20–59 mg/l), N (%)	6 (28.6)	7 (38.9)	
Severely elevated (60–300 mg/l), N (%)	11 (52.4)	10 (55.6)	
D-dimer, mg/l, median (range)^a^	1.35 (0.36–22.9)	0.92 (0.31–19.4)	0.26
Increased (>0.50), N (%)	13 (92.9)	9 (69.2)	0.28
Ferritin, μg/l, median (range)	605 (45–3767)	716 (30–1700)	0.73
Increased (>355), N (%)	17 (77.3)	14 (73.7)	1.00
LDH, U/l, median (range)	272 (143–555)	286 (164–454)	0.99
Increased (>255), N (%)	12 (54.5)	14 (73.7)	0.35
ALAT, U/l, median (range)	26 (10–164)	45 (19–110)	0.087
Increased (>50), N (%)	5 (22.7)	7 (36.8)	0.52
Total bilirubin, μmol/l, median (range)	7.5 (4–24)	9 (4–28)	0.79
Increased (>25), N (%)	0 (0.0)	1 (5.3)	0.94
Decreased (<5), N (%)	1 (4.5)	1 (5.3)	1.00
Creatinine, μmol/l, median (range)	75 (46–123)	77 (41–132)	0.89
Increased (>105), N (%)	4 (18.2)	2 (10.5)	0.80
Decreased (<45), N (%)	0 (0.0)	1 (5.3)	0.94
Infiltrates on chest X-ray, N (%)			
Yes	17 (77.3)	15 (78.9)	1.00
No	4 (18.2)	4 (21.1)	1.00
Not investigated	1 (4.5)	0 (0.0)	1.00

*All the laboratory findings were obtained from the time of inclusion in the study. Threshold values of the biochemical data are based on guidelines provided to the healthcare services in the North Denmark Region*.

a *Patients were excluded from the statistical analysis because of undetectable or missing values. For PaO_2_, the statistical analysis was based on 18 rectal positive patients and 16 rectal negative patients. When measuring PaO_2_, 61.1% rectal positive patients and 43.8% rectal negative patients received oxygen supply. When measuring saturation, 50.0% rectal positive patients and 52.6% rectal negative patients received oxygen supply. The oxygen supply ranged from 1 to 15 l. For CRP, the statistical analysis was based on 21 rectal positive patients and 18 rectal negative patients. For D-dimer, the statistical analysis was based on 14 rectal positive patients and 13 rectal negative patients. CI, Confidence interval; kPa, Kilopascal; PaO_2_, The partial pressure of oxygen in arterial blood; CRP, C-reactive protein; LDH, Lactate dehydrogenase; ALAT, Alanine aminotransferase*.

### Duration of SARS-CoV-2 Rectal Shedding Among Adults and Children

**Figure 1:**
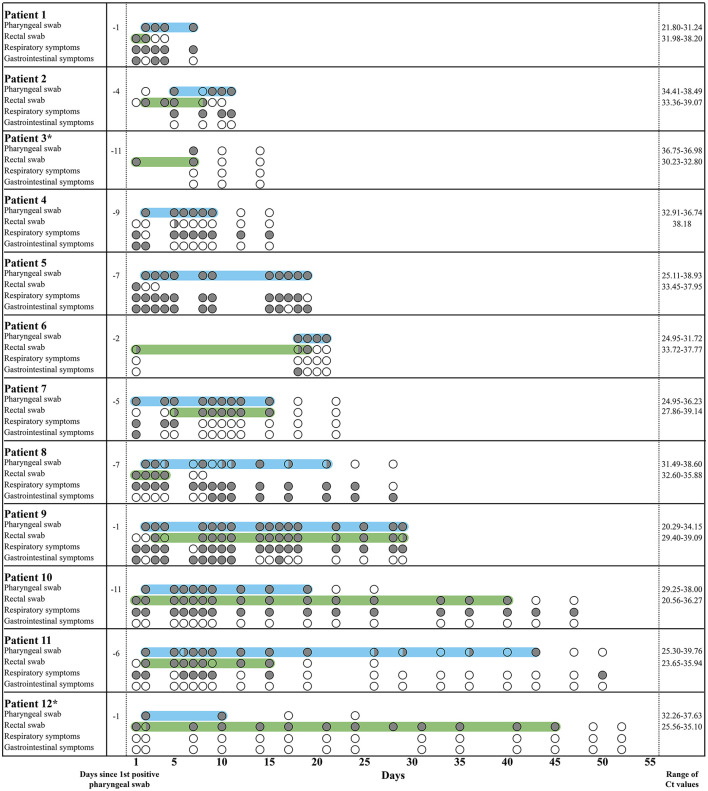
Pharyngeal and rectal swab results from COVID-19 patients followed for more than 6 days. In the rows with pharyngeal and rectal swabs, a gray circle (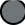) illustrates a positive result, a transparent circle (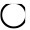) illustrates a negative result, and a half-filled circle (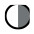) illustrates an inconclusive result, where only one of the duplicates was positive. In the rows with respiratory and gastrointestinal symptoms, a gray circle (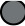) illustrates the presence of the symptoms, and a transparent circle (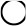) illustrates that the symptoms were not experienced. Children are marked by *. The light blue area marks the period where the pharyngeal swabs were positive, while the light green area marks the period where the rectal swabs were positive. Respiratory symptoms include cough, sore throat, sneeze, dyspnea, and colored sputum. Gastrointestinal symptoms include nausea, vomit, stomach ache, and diarrhea.

The mean duration of rectal positivity until two consecutive negative rectal swabs was 13.7 days. The longest duration of rectal SARS-CoV-2 shedding was 45 days from inclusion (Figure 1). Two of seven participants (28.6%), who were followed for more than 6 days, continued to test positive in their rectal swabs after their pharyngeal swabs turned negative up to 29 days after testing (Figure 1). Ct-values ranged from 20.29 to 39.76 in the pharyngeal swabs and 20.56–39.14 in the rectal swabs (Figure 1; Supplementary Figure 1). In most participants, Ct-values were higher for the rectal swabs compared to the pharyngeal swabs, which may indicate a lower viral load in the rectal swabs. However, in patient 3, 10, and 12, Ct-values were lower for the rectal swabs. Patient 1, 2, 5, 6, and 9 were finalized in the study before a negative conversion of the samples was obtained due to discharge or transfer to other departments (Figure 1). No correlation was seen between the presence of SARS-CoV-2 in rectal swabs and the experience of gastrointestinal symptoms (Figure 1).

## Discussion

Rectal shedding of SARS-CoV-2 was observed in 28 of 52 (53.8%) COVID-19 positive adults and children with a duration of up to 45 days from inclusion. Notably, prolonged rectal shedding after negative conversion of pharyngeal swabs was only observed in two of seven (28.6%) COVID-19 positive adults and children, who were followed for more than 6 days. The rectal shedding proceeded up to 29 days after the pharyngeal shedding had stopped. The hospitalized adult rectal positive patients and rectal negative patients were comparable regarding demographic, medical, and biochemical information.

In previous studies, prolonged rectal shedding after negative conversion of respiratory samples has been observed in up to 78.0% of the COVID-19 patients (25, 27, 38), whereas we only observed this for two of our patients. This discrepancy may be explained by several factors; first, we were not able to follow all our patients until a negative conversion of pharyngeal and rectal swabs occurred, leading to a likely underestimation of prolonged rectal shedding. Second, there may be changes or differences in treatment strategies between countries and over time, which could have an impact on rectal SARS-CoV-2 shedding. For instance, antiviral treatment has been shown to be positively correlated with the presence of SARS-CoV-2 in feces (25). We did not, however, in our study observe any correlation between antiviral treatment and duration or prevalence of rectal shedding. Finally, we included patients at very different time points during their disease course, making it difficult to completely map out when rectal SARS-CoV-2 was predominantly present.

There has been an ongoing debate on whether rectal SARS-CoV-2 shedding is linked to disease severity. Our study showed no correlation, which is in line with the results of Chen et al. (27). Another study (39), however, showed a positive correlation between rectal shedding and disease severity. The discrepancy between the studies may be related to the different parameters used to assess the severity of the disease. Therefore, a definitive correlation between rectal shedding of SARS-CoV-2 and disease severity has not yet been established, but it appears that SARS-CoV-2 can be present in the intestines without necessarily affecting the severity of the disease. This is supported by the high Ct-values for the rectal swabs compared with the pharyngeal swabs, which may indicate a low viral load in the rectal swabs. Notably, Ct-values are not equivalent to viral load but are only an indicator, as the Ct-values are also affected by the procedure of the sample collection. However, it is still unknown whether the presence of SARS-CoV-2 in the intestines has long-term consequences for the infected individuals, such as an influence on the gut function or the immune responses. Overall, the clinical importance of rectal SARS-CoV-2 shedding remains unknown, and future studies investigating the possible long-term consequences are needed.

Although SARS-CoV-2 has been detected in the intestines of infected individuals, the infectious potential continues to be undetermined. A few studies have been able to isolate active SARS-CoV-2 from the feces of infected individuals (24, 26) and observe active viral replication in rectal tissues (40). Therefore, evidence suggests that the virus is actively replicating in the intestines and is not just non-infectious leftovers from the respiratory tract. However, evidence of replication in the intestines is not synonymous with the virus from feces being infectious. Zang et al. (41) showed that SARS-CoV-2 could be inactivated *in vitro* by simulated colonic fluid. Thus, the virus may be inactivated relatively fast when released to the intestinal lumen, and the infectious risk of the virus from feces may be of little concern.

Despite the uncertainty concerning the clinical importance and infectious potential of rectal SARS-CoV-2 shedding, the observation of rectal shedding has proven advantageous in SARS-CoV-2 testing of sewage samples, where it is possible to monitor potential outbreaks of infection in the community (42).

There are some limitations in our study that need to be addressed. First, a fraction of the rectal samples was collected by the participants themselves, leading to the risk of incorrect collection. However, to compensate for this, thorough instructions were given before sample collection. Another limitation is that participants were included at different stages in their disease course, which may have had an impact on the number of rectal positive participants identified. Nonetheless, no correlation was observed between the time of inclusion and the rectal positivity. In addition, not all participants were followed until two consecutive negative pharyngeal and rectal swabs were obtained. Furthermore, the patients with the most severe disease course may have been incapable of giving consent and could therefore not be included in the study, which may have affected the study's results. Finally, the number of COVID-19 positive participants in each group was low and investigating a larger cohort would provide more information about the duration of rectal shedding, as well as its clinical significance.

Nonetheless, the present study has strengths. First, we applied regular collection of both pharyngeal and rectal samples with parallel reporting of symptoms. Furthermore, we obtained detailed demographic and clinical information about the individual participants through questionnaires and medical records.

In conclusion, this study provided evidence of rectal SARS-CoV-2 shedding in Danish COVID-19 patients. However, as opposed to previous studies, we only observed prolonged rectal shedding in a few COVID-19 patients. The clinical importance of rectal SARS-CoV-2 shedding appears to be minimal, however, long-term consequences and the infectious potential of rectal shedding remain to be determined.

## Data Availability Statement

The raw data supporting the conclusions of this article will be made available by the authors, without undue reservation.

## Ethics Statement

The studies involving human participants were reviewed and approved by North Denmark Region Committee on Health Research Ethics (N-20200036). Written informed consent to participate in this study was provided by the participants' legal guardian/next of kin.

## Author Contributions

JH-J, CB-N, A-MJ, SSh, ML, KK, MD, BP, LA, SH, PL, and SSø planned and designed the study. JH-J, SSh, and JV recruited participants to the study. Data extraction was performed by JH-J, KK, and SSh. Data analyses were performed by JH-J, CB-N, LR, A-MJ, and SSø. JH-J and SSø drafted the initial manuscript, while all authors contributed to finalizing the manuscript. All authors read and approved the final manuscript.

## Funding

This study was funded by the Novo Nordisk Foundation under grant NNF20SA0062182. The funding source was not involved in study design, sample collection, analysis, interpretation of data, or preparation of the manuscript.

## Conflict of Interest

The authors declare that the research was conducted in the absence of any commercial or financial relationships that could be construed as a potential conflict of interest.

## Publisher's Note

All claims expressed in this article are solely those of the authors and do not necessarily represent those of their affiliated organizations, or those of the publisher, the editors and the reviewers. Any product that may be evaluated in this article, or claim that may be made by its manufacturer, is not guaranteed or endorsed by the publisher.
