# Supplementary material for: The Prevalence and Clinical Implications of Rectal SARS-CoV-2 Shedding in Danish COVID-19 Patients and the General Population
Source: Front Med (Lausanne). 2022 Jan 13;8:804804. doi: 10.3389/fmed.2021.804804 (PMC8792906; doi:10.3389/fmed.2021.804804)
Supplement: Supplementary file 1 [file Table_1.docx]

Supplementary Material

# Supplementary Data

**Supplementary Table 1: Demographic and clinical characteristics of hospitalized COVID-19 positive and COVID-19 negative adult patients.** ^a^Alcohol consumption was assessed according to the recommendations made by the Danish Health Authority about the low-risk limit for women (7 units per week) and men (14 units per week). Intestinal disease includes Crohn’s disease, diverticulitis, steatosis, bowel cancer, diaphragmatic hernia, gastric bypass, and intestinal resection. Cardiovascular disease includes hypertension, pulmonary hypertension, transient cerebral ischemia, ischemic heart disease, non-STEMI coronary thrombosis, atrial fibrillation, hypercholesterolemia, arterial sclerosis, femoral bypass surgery, cardiac insufficiency, cerebral apoplexy, normal pressure hydrocephalus, aortic stenosis, aortic aneurysm, mechanic heart valves, and 3rd degree AV block with subsequent pacemaker implantation. Pulmonary disease includes asthma, chronic obstructive pulmonary disease, sleep apnea, partial lung resection, and slightly impaired lung function. Vaccination status was self-reported. ^b^Patients were excluded from the statistical analysis because of undetectable or missing values. For vaccination status statistical analyses were based on 23 COVID-19 positive patients and 11 COVID-19 negative patients. CI: Confidence interval, BMI: Body Mass Index, COPD: Chronic Obstructive Pulmonary Disease, NA: Not available.

| **Demographics** | **COVID-19  N = 41 (78.8 %)** | **COVID-19 negative  N = 11 (21.2 %)** | **P-value** |
| --- | --- | --- | --- |
| Age, years, median (CI) | 70 (65.3-72.2) | 73 (58.0-81.6) | 0.56 |
| Gender, N (%) | | | |
| - Male | 27 (65.9) | 6 (54.5) | 0.73 |
| - Female | 14 (34.1) | 5 (45.5) | 0.73 |
| BMI, mean (CI) | 28.8 (27.2-30.4) | 28.5 (23.6-33.4) | 0.92 |
| Lives in a nursing home, N (%) | 2 (4.9) | 0 (0.0) | 1.00 |
| Smoking, N (%) | | | |
| - Yes | 3 (7.3) | 2 (18.2) | 0.61 |
| - No | 16 (39.0) | 2 (18.2) | 0.35 |
| - Former | 22 (53.7) | 7 (63.6) | 0.80 |
| Alcohol consumption, N (%) | | | |
| - More units/week than recommended^a^ | 4 (9.8) | 0 (0.0) | 0.66 |
| Occupation, N (%) | | | |
| - Healthcare | 4 (9.8) | 1 (9.1) | 1.00 |
| - Educational sector | 0 (0.0) | 0 (0.0) | NA |
| - Eldercare | 0 (0.0) | 0 (0.0) | NA |
| - Children and adolescents | 0 (0.0) | 0 (0.0) | NA |
| - Retired | 28 (68.3) | 9 (81.8) | 0.61 |
| - Other | 9 (22.0) | 1 (9.1) | 0.60 |
| **Clinical characteristics** | **COVID-19  N = 41 (78.8 %)** | **COVID-19 negative  N = 11 (21.2 %)** | **P-value** |
| Intestinal disease, N (%) | 6 (14.6) | 1 (9.1) | 1.00 |
| Risk factors, N (%) | | | |
| - Cardiovascular disease | 27 (65.9) | 9 (81.8) | 0.52 |
| - Hypertension | 24 (58.5) | 9 (81.8) | 0.28 |
| - Pulmonary disease | 15 (36.6) | 6 (54.5) | 0.46 |
| - Asthma | 5 (12.2) | 3 (27.3) | 0.45 |
| - COPD | 8 (19.5) | 3 (27.3) | 0.89 |
| - Severe overweight (BMI > 30) | 18 (43.9) | 3 (27.3) | 0.51 |
| - Type 1 or 2 diabetes | 7 (17.1) | 2 (18.2) | 1.00 |
| - Cancer | 9(22.0) | 1 (9.1) | 0.60 |
| Symptoms, N (%) | | | |
| - Cough | 35 (85.4) | 9 (81.8) | 1.00 |
| - Dyspnea | 30 (73.2) | 9 (81.8) | 0.84 |
| - Fever | 24 (58.5) | 4 (36.4) | 0.33 |
| - Gastrointestinal symptoms | 21 (51.2) | 5 (45.5) | 1.00 |
| - Nausea | 12 (29.3) | 1 (9.1) | 0.33 |
| - Vomiting | 5 (12.2) | 1 (9.1) | 1.00 |
| - Stomach ache | 11 (26.8) | 2 (18.2) | 0.84 |
| - Diarrhea | 17 (41.5) | 4 (36.4) | 1.00 |
| Sore throat | 16 (39.0) | 3 (27.3) | 0.71 |
| Affected taste or smell | 16 (39.0) | 1 (9.1) | 0.13 |
| Headache | 13 (31.7) | 2 (18.2) | 0.61 |
| - No symptoms | 0 (0.0) | 0 (0.0) | NA |
| Vaccination, N (%)^b^ | | | |
| - Vaccinated with first dose | 1 (4.3) | 0 (0.0) | 1.00 |
| - Fully vaccinated | 0 (0.0) | 0 (0.0) | NA |
| Rectal swab, N (%) | | | |
| - Positive rectal swab | 22 (53.7) | 0 (0.0) | **0.0043** |

**Supplementary Table 2: Demographic and clinical characteristics of COVID-19 positive and COVID-19 negative children.** NA: Not available.

| **Demographics** | **COVID-19  N = 2 (18.2 %)** | **COVID-19 negative  N = 9 (81.1 %)** |
| --- | --- | --- |
| Age, years, median (range) | 2.5 (0-5) | 1 (0-8) |
| Gender, N (%) | | |
| - Male | 1 (50.0) | 4 (44.4) |
| - Female | 1 (50.0) | 5 (55.6) |
| Occupation, N (%) | | |
| - Day nursery | 0 (0.0) | 0 (0.0) |
| - Daycare | 0 (0.0) | 2 (22.2) |
| - Kindergarten | 0 (0.0) | 1 (11.1) |
| - Primary school | 1 (50.0) | 2 (22.2) |
| - Continuation school | 0 (0.0) | 0 (0.0) |
| - Taken care of at home or private | 1 (50.0) | 3 (33.3) |
| - Other | 0 (0.0) | 1 (11.1) |
| **Clinical characteristics** | **COVID-19 N = 2 (18.2 %)** | **COVID-19 negative N = 9 (81.1 %)** |
| Hospitalized, N (%) | 1 (50.0) | 2 (22.2) |
| Intestinal disease, N (%) | 0 (0.0) | 0 (0.0) |
| Risk factors, N (%) | | |
| - Cardiovascular disease | 0 (0.0) | 0 (0.0) |
| - Pulmonary disease | 0 (0.0) | 0 (0.0) |
| - Type 1 or 2 diabetes | 0 (0.0) | 0 (0.0) |
| - Cancer | 0 (0.0) | 0 (0.0) |
| Risk factors, N (%) | | |
| - Fever | 1 (50.0) | 5 (55.6) |
| - No symptoms | 1 (50.0) | 2 (22.2) |
| - Cough | 0 (0.0) | 4 (44.4) |
| - Gastrointestinal symptoms | 0 (0.0) | 3 (33.3) |
| - Nausea | 0 (0.0) | 0 (0.0) |
| - Vomiting | 0 (0.0) | 1 (11.1) |
| - Stomach ache | 0 (0.0) | 2 (22.2) |
| - Diarrhea | 0 (0.0) | 1 (11.1) |
| - Sore throat | 0 (0.0) | 2 (22.2) |
| - Dyspnea | 0 (0.0) | 0 (0.0) |
| - Headache | 0 (0.0) | 1 (11.1) |
| - Altered taste or smell | 0 (0.0) | 0 (0.0) |
| Rectal swab, N (%) | | |
| - Positive rectal swab | 2 (100.0) | 0 (0.0) |

**Supplementary Table 3: Demographic and clinical characteristics of non-hospitalized COVID-19 positive and COVID-19 negative adult participants.** ^a^Alcohol consumption was assessed according to the recommendations made by the Danish Health Authority about the low-risk limit for women (7 units per week) and men (14 units per week). Intestinal disease includes Crohn’s disease, diverticulitis, colorectal cancer, gastric bypass, irritable colon, ileorectal anastomosis, total colectomy, rectocele, and use of ostomy. Cardiovascular disease includes hypertension, myocardial infarction, ischemic heart disease, hypercholesterolemia, atrial fibrillation, acute myocardial infarction, dilated cardiomyopathy, aortic stenosis, chronic anemia, aortic insufficiency, palpitation, and coronary thrombosis with subsequent balloon angioplasty. Pulmonary disease includes asthma, chronic obstructive pulmonary disease, pulmonary embolism, lung cancer, pneumonectomy, and emphysema. Vaccination status was self-reported. CI: Confidence interval, BMI: Body Mass Index, COPD: Chronic Obstructive Pulmonary Disease, NA: Not available

| **Demographics** | **COVID-19  N = 9 (4.3 %)** | **COVID-19 negative  N = 202 (95.7 %)** |
| --- | --- | --- |
| Age, years, median (CI) | 50 (38.4-57.2) | 58.5 (55.7-59.5) |
| Gender, N (%) | | |
| - Male | 2 (22.2) | 78 (38.6) |
| - Female | 7 (77.8) | 124 (61.4) |
| BMI, median (CI) | 25.3 (23.0-34.8) | 26.9 (27.0-28.6) |
| Live in a nursing home, N (%) | 0 (0.0) | 0 (0.0) |
| Smoking, N (%) | | |
| - Yes | 0 (0.0) | 32 (15.9) |
| - No | 8 (88.9) | 118 (58.7 |
| - Former | 1 (11.1) | 51 (25.4) |
| Alcohol consumption, N (%) | | |
| - More units/week than recommended^a^ | 1 (14.3) | 27 (13.8) |
| Occupation, N (%) | | |
| - Other | 4 (44.4) | 45 (22.5) |
| - Retired | 1 (11.1) | 74 (37.0) |
| - Healthcare | 3 (33.3) | 41 (20.5) |
| - Eldercare | 1 (11.1) | 10 (5.0) |
| - Unemployed/on medical leave | 0 (0.0) | 12 (6.0) |
| - Children and adolescents | 0 (0.0) | 11 (5.5) |
| - Student | 0 (0.0) | 5 (2.5) |
| - Educational sector | 0 (0.0) | 2 (1.00) |
| Reason for test, N (%) | | |
| - Visit to the doctor or hospital | 0 (0.0) | 90 (46.4) |
| - Contact | 4 (44.4) | 13 (6.7) |
| - Symptoms | 3 (33.3) | 52 (26.8) |
| - Known positive | 2 (22.2) | 0 (0.0) |
| - Screening (Healthcare workers) | 0 (0.0) | 19 (9.8) |
| - Screening (By own request) | 0 (0.0) | 13 (6.7) |
| - Work | 0 (0.0) | 1 (0.5) |
| - Travel | 0 (0.0) | 3 (1.5) |
| - Visit to vulnerable people | 0 (0.0) | 3 (1.5) |
| **Clinical characteristics** | **COVID-19 N = 9 (4.3 %)** | **COVID-19 negative N = 202 (95.7 %)** |
| Intestinal disease, N (%) | 0 (0.0) | 37 (18.3) |
| Risk factors, N (%) | | |
| - Severe overweight (BMI > 30) | 3 (33.3) | 56 (27.9) |
| - Cardiovascular disease | 1 (11.1) | 53 (26.2) |
| - Hypertension | 1 (11.1) | 34 (16.8) |
| - Pulmonary disease | 1 (11.1) | 20 (9.9) |
| - Asthma | 1 (11.1) | 9 (4.5) |
| - COPD | 0 (0.0) | 13 (6.4) |
| - Cancer | 1 (11.1) | 16 (7.9) |
| - Type 1 or 2 diabetes | 0 (0.0) | 14 (6.9) |
| Symptoms, N (%) | | |
| - Cough | 7 (77.8) | 45 (23.6) |
| - Fever | 6 (66.7) | 24 (12.6) |
| - Sore throat | 6 (66.7) | 51 (26.7) |
| - No symptoms | 2 (22.2) | 111 (58.1) |
| - Headache | 6 (66.7) | 51 (26.7) |
| - Gastrointestinal symptoms | 4 (44.4) | 29 (15.2) |
| - Nausea | 4 (44.4) | 17 (8.9) |
| - Vomiting | 1 (11.1) | 6 (3.1) |
| - Stomach ache | 0 (0.0) | 13 (6.8) |
| - Diarrhea | 1 (11.1) | 13 (6.8) |
| - Dyspnea | 3 (33.3) | 25 (13.1) |
| - Altered taste or smell | 3 (33.3) | 11 (5.8) |
| Vaccinated, N (%) | | |
| - Vaccinated with first dose | 0 (0.0) | 10 (5.2) |
| - Fully vaccinated | 0 (0.0) | 4 (2.1) |
| Rectal swab, N (%) | | |
| - Positive rectal swab | 4 (44.4) | 0 (0.0) |

**Supplementary Table 4:** **WHO clinical progression score of hospitalized rectal positive and rectal negative adult patients.** NIV: Non-invasive ventilation, ECMO: Extra Corporeal Membrane Oxygenation.

| **WHO clinical progression score, N (%) (at inclusion/at the patient’s worst)** | **Positive rectal swab**  **N = 22 (53.7 %)** | **Negative rectal swab**  **N = 19 (46.3 %)** | **P-value** |
| --- | --- | --- | --- |
| - Score 4 (No oxygen therapy) | 11 (50.0) / 7 (31.8) | 12 (63.2) / 3 (15.8) | 0.51 / 0.36 |
| - Score 5 (Oxygen by mask or nasal prongs) | 10 (45.5) / 9 (40.9) | 7 (36.8) / 12 (63.2) |  |
| - Score 6 (Oxygen by NIV or high flow) | 1 (4.5) / 2 (9.1) | 0 (0.0) / 3 (15.8) |  |
| - Score 7 (Intubation and mechanical ventilation, pO_2_/FiO_2_ ≥150 or SpO_2_/FiO_2_≥200) | 0 (0.0) / 0 (0.0) | 0 (0.0) / 0 (0.0) |  |
| - Score 8 (Mechanical ventilation pO_2_/FiO_2_ <150 (SpO_2_/FiO_2_<200) or vasopressors) | 0 (0.0) / 2 (9.1) | 0 (0.0) / 0 (0.0) |  |
| - Score 9 (Mechanical ventilation pO_2_/FiO_2_ <150 and vasopressors, dialysis, or ECMO) | 0 (0.0) / 0 (0.0) | 0 (0.0) / 0 (0.0) |  |
| - Score 10 (Dead) | 0 (0.0) / 2 (9.1) | 0 (0.0) / 1 (5.3) |  |

**Supplementary Figure 1:** **Ct-values of pharyngeal and rectal swabs from participants followed for more than six days.**
